# Artificial gut and the applications in poultry: A review

**DOI:** 10.1016/j.aninu.2021.12.010

**Published:** 2022-03-26

**Authors:** Nishchal K. Sharma, Shu-Biao Wu, Natalie K. Morgan, Tamsyn M. Crowley

**Affiliations:** a School of Environmental and Rural Science, University of New England, Armidale, NSW, 2351, Australia; b Poultry Hub Australia, University of New England, Armidale, NSW, 2351, Australia; c IMPACT, School of Medicine, Deakin University, Geelong, Victoria, 3217, Australia

**Keywords:** Artificial gut, Gastrointestinal tract, In vitro experiment, Poultry, Simulated gut

## Abstract

Artificial gut models including both the gastric and intestinal phases have been used in poultry research for decades to predict the digestibility of nutrients, the efficacy of feed enzymes and additives, and caecal fermentation. However, the models used in the past are static and cannot be used to predict interactions between the feed, gut environment and microbiome. It is imperative that a standard artificial gut model for poultry is established, to enable these interactions to be examined without continual reliance on animals. To ensure the validity of an artificial model, it should be validated with in vivo studies. This review describes current practices in the use of artificial guts in research, their importance in poultry nutrition studies and highlights an opportunity to develop a dynamic gut model for poultry to reduce the number of in vivo experiments.

## 1 Introduction

In vivo experiments are commonly used in poultry health and nutrition research to examine growth performance, health, nutrient utilisation, and other physiological status of animals in response to nutritional, disease and environmental treatments. In vivo poultry research trials are reliable, and have led to substantial advances in poultry science over the last few decades. However, these trials involve utilisation of large numbers of animals, which is an ethical concern. Moreover, conducting in vivo trials is time-consuming and expensive, and requires accessibility to specialised facilities for rearing chickens and manufacturing feed, alongside technical expertise and animal ethics approval from an official body. In vitro methods on the other hand are simple, less expensive and time-consuming, and do not rely on use of animals. Simulating in vivo conditions using in vitro techniques, such as artificial guts, allows for reduction and replacement of animals when testing products and concepts. This review will discuss current practices and future use of in vitro assays that mimic the chicken gastrointestinal (GI) tract, for use in poultry nutrition research.

## 2 The artificial gut

The practice of using a simulated gut in poultry research was first employed more than 40 years ago (Sakamoto et al., 1980), and many in vitro nutrition studies have been undertaken since. Examples of in vitro experiments that have used artificial gut models are summarised in Table 1, Table 2, Table 3, Table 4, Table 5, Table 6.

**Table 1:** Artificial guts used to estimate digestibility of nutrients in poultry.

Measurement	Simulated portion of the gut	Assay condition								Simulated animal/species	Reference
		Proventriculus/Gizzard (gastric phase)				Small intestine (SI phase)					
		Commercial enzyme/chemical	Incubation time, min	T, °C	pH	Commercial enzyme/chemical	Incubation time, min	T, °C	pH		
Crude protein and dry matter	Gastric and SI phases		240	37	NA		240	37	NA	White Leghorn hen	Sakamoto et al. (1980)
Crude protein and dry matter	Gastric and SI phases		60, 120, 180, 240	37	NA		60 (after 240 min in gastric phase), 120, 180, 240	37	6.5, 6.6, 6.7, 6.8, 6.9	Broiler chicken	Clunies and Leeson (1984)
Starch	Gastric and SI phases	Pepsin, HCl	30	37	NA	Pancreatin, amyloglucosidase, invertase	15–360	37	5.2		Weurding et al. (2001)
Starch	Gastric and SI phases	Pepsin, HCl	30	41	2.5	Pancreatin, amyloglucosidase, invertase	15–360	41	5.6	Broiler chicken	Ebsim (2013)
Starch	Gastric and SI phases	Pepsin, HCl	30	42	2.5		15–240	41	5.6	Broiler chicken	Karunaratne et al. (2018a)
Starch	Gastric and SI phases	Pepsin, HCl	30	42	2.5		15–240	41	5.6	Broiler chicken	Karunaratne et al. (2018b)
Crude protein	Gastric and SI phases	Pepsin, HCl	30	41	NA	Pancreatin	180	41	7.5	Broiler chicken	Bryan et al. (2018)
Crude protein	Gastric and SI phases	Pepsin, HCl	30	41	NA	Pancreatin	180	41	7.5	Broiler chicken	Bryan et al. (2019)
Crude protein	Gastric and SI phases	Pepsin, HCl	30	41	NA	Pancreatin	180	41	7.5	Broiler chicken	Bryan and Classen (2020)

SI = small intestine; T = temperature.

**Table 2:** Artificial guts used to estimate energetic values of ingredients and feeds for poultry.

Simulated portion of the gut	Assay condition								Simulated animal/species	Reference
	Proventriculus/Gizzard (gastric phase)				Small intestine (SI phase)					
	Commercial enzyme/chemical	Incubation time, min	T, °C	pH	Commercial enzyme/chemical	Incubation time, min	T, °C	pH		
Gastric and SI phases		240	37	NA		240	37	7.2	Adult chicken rooster	Clunies et al. (1984)
Gastric and SI phases	Pepsin (20 mg), HCl	240	37	4.1	Pancreatin (40 mg), bile salts (15 mg), enterokinase (2.5 mg)	360	37	7.0–7.1	Adult chicken rooster	Valdes and Leeson (1992)
Gastric and SI phases	Pepsin, HCl	120	39	2.0	Pancreatin	420	39	6.8	Adult chicken rooster	Losada et al. (2009)
Gastric and SI phases	Pepsin, HCl	120	39	2.0	Pancreatin	420	39	6.8	Adult chicken rooster	Losada et al. (2010)
Gastric and SI phases	Pepsin, HCl	120	41	2.0		240	41	6.8	Broiler chicken	Yegani et al. (2013)
Gastric and SI phases	Pepsin, HCl	240	41	2.0	Amylase, trypsin, chymotrypsin	30 (upper SI), 7.5 h (lower SI)	41	6.5 (upper SI), 8.1 (lower SI)	Chinese yellow rooster	Zhao et al. (2014)
Gastric and SI phases	Pepsin, HCl	240	41	2.0	Amylase, trypsin, chymotrypsin	30 (upper SI), 7.5 h (lower SI)	41	6.5 (upper SI), 8.1 (lower SI)	Peking duck	Zhang et al. (2019)

SI = small intestine; T = temperature.

**Table 3:** Artificial guts used to assess the efficacy of carbohydrases and protease for poultry.

Simulated portion of the gut	Assay condition								Simulated animal/species	Reference
	Proventriculus/Gizzard (gastric phase)				Small intestine (SI phase)					
	Commercial enzyme/chemical	Incubation time, min	T, °C	pH	Commercial enzyme/chemical	Incubation time, min	T, °C	pH		
Gastric and SI phases	Pepsin, HCl	45	40	2.5	Pancreatin, Na Bicarbonate	120	40	6.3–6.7	Broiler chicken	Bedford and Classen (1993)
Crop, gastric and SI phases	Pepsin, HCl	30 (crop) + 45 (gizzard)	40	3.0	Pancreatin	60	40	6.5	Broiler chicken	Tervila-Wilo et al. (1996)
Crop, gastric and SI phases	–	–	–	3.0	–	–	–	7.0, 7.5	Broiler chicken	Ao et al. (2008)
		Crop pH 6.5								
Crop, gastric and SI phases	Pepsin, HCl	30 (crop, pH not adjusted) + 45 (gizzard)	40	3.0	Pancreatin	60	40	6.5	Broiler chicken	Ao et al. (2010)
Crop, gastric and SI phases	Pepsin, HCl	30 (crop, pH 6.0) + 45 (proventriculus/gizzard)	40	3.0	Pancreatin, Na Bicarbonate	60	40	6.5	Broiler chicken	Fengying et al. (2011)
Crop, gastric and SI phases	Pepsin, HCl	30 (crop, pH 5.2) + 45 (gizzard)	42 ± 1	1.4–2.0	Pancreatin	120	42 ± 1	6.4–6.8	Broiler chicken	Suresh et al. (2019)
Gastric and SI phases	Pepsin, HCl	20	42	3.5	Pancreatin, Sodium bicarbonate	80	42	6.0	Broiler chicken	Morgan et al. (2019)

SI = small intestine; T = temperature.

**Table 4:** Artificial guts used to assess the effects of phytase for poultry.

Simulated portion of the gut	Assay condition								Simulated animal/species	Reference
	Proventriculus/Gizzard (gastric phase)				Small intestine (SI phase)					
	Commercial enzyme/chemical	Incubation time, min	T, °C	pH	Commercial enzyme/chemical	Incubation time, min	T, °C	pH		
Crop, gastric and SI phases	Pepsin, HCl	30 (crop, pH 5.25) + 45 (gizzard)	40	NA	Pancreatin	240	40	NA	Turkey	Zyla et al. (1995)
Crop, gastric and SI phases	Pepsin, HCl	30 (crop) + 45 (gizzard)	40	NA	Pancreatin	240	40	NA	Turkey	Zyla et al. (1996)
Crop, gastric and SI phases	Pepsin, HCl	30 (crop, pH 5.8) + 45 (gizzard)	40	2.75	Pancreatin	240, 60	40	6.1	Broiler chicken	Zyla et al. (1999)
Crop, gastric and SI phases	Pepsin, HCl	30 (crop, pH 5.8) + 45 (gizzard)	40	2.75	Pancreatin	240	40	6.1	Broiler chicken	Zyla et al. (2000)
Crop, gastric and SI phases	Pepsin, HCl	30 (crop, pH- 5.5) + 45 (gizzard)	40	2.5	Pancreatin	60, 240	40	6.0	Broiler chicken	Lan et al. (2010)
Gastric and SI phases	Pepsin, HCl	42	40	2.75–3.25	Pancreatin, Sodium bicarbonate	60	40	6.3–6.7	Broiler chicken	Walk et al. (2012a)
Exp 1-Gastric and SI phases	Pepsin, HCl	42 (Exp. 1)	40	2.75–3.25	Pancreatin, Sodium bicarbonate	60	40	6.3–6.7	Broiler chicken	Walk et al. (2012b)
Exp 2- Gastric phase		5, 10, 20 (Exp. 2)								
Gastric and SI phases	Pepsin, HCl	20	41	3.5–4.5	Pancreatin, Sodium bicarbonate	80	41	6.3–6.7	Broiler chicken	Morgan et al. (2014)
Crop, gastric and SI phases	Pepsin, HCl	30 (crop, pH 5.0) + 45 (gizzard)	40	3.0	Pancreatin	60	40	6.10	Broiler chicken	Menezes-Blackburn et al. (2015)
Gastric and SI phases	Pepsin, HCl	42	40	2.5	Pancreatin, Sodium bicarbonate	60	40	6.3–6.7	Broiler chicken	Farhadi et al. (2019)

SI = small intestine; T = temperature.

**Table 5:** Artificial guts used to evaluate the efficacy of adsorbents against mycotoxins.

Simulated portion of the gut	Assay condition								Simulated animal/species	Reference
	Proventriculus/Gizzard (gastric phase)				Small intestine (SI phase)					
	Commercial enzyme/chemical	Incubation time, min	T, °C	pH	Commercial enzyme/chemical	Incubation time, min	T, °C	pH		
Crop, gastric and SI phases	Pepsin, HCl	30 (crop, pH 5.2) + 45 (proventriculus)	40	1.4–2.0	Pancreatin	120	40	6.4–6.8	Broiler chicken	Solís-Cruz et al. (2017)
Crop, gastric and SI phases	Pepsin, HCl	30 (crop, pH 5.2) + 45 (proventriculus)	40	1.4–2.0	Pancreatin	120	40	6.4–6.8	Broiler chicken	Zavala-Franco et al. (2018)
Crop, gastric and SI phases	Pepsin, HCl	60, 120 (crop, pH 4.5) + 30 (proventriculus)	40	2.5	Pancreatin	60, 120	40	6.5	Broiler chicken	Tso et al. (2019)
Crop, gastric and SI phases	Pepsin, HCl	60 (crop, pH 4.5–5.3) + 90 (proventriculus)	40	1.9–3.7	Pancreatin, Sodium bicarbonate, bile salt	120	40	5.3–7.5	Broiler chicken	Kolawole et al. (2019)

SI = small intestine; T = temperature.

**Table 6:** Artificial guts used to determine the efficacy of feed additives and estimation of caecal fermentation.

Simulated portion of the gut	Assay condition								Simulated animal/species	Reference
	Proventriculus/Gizzard (gastric phase)				Small intestine (SI phase)					
	Commercial enzyme/chemical	Incubation time, min	T, °C	pH	Commercial enzyme/chemical	Incubation time, min	T, °C	pH		
Crop, gastric and SI phases	Pepsin, HCl	30 (crop, pH 4.5) + 15 (proventriculus, pH 4.4) + 90 (gizzard)	41.4	2.6	Pancreatin	90	41.4	6.2	Broiler chicken	Chang and Chen (2000)
						15 (caeca)		6.3 (caeca)		
Crop, gastric and SI phases	Pepsin, HCl	45 (crop, pH 4.6) + 90 (proventriculus/gizzard)	41.4	2.5	Pancreatin	150	41.4	6.2	Broiler chicken	Wali and Beal. (2011)
						15 (caeca)				
Crop, gastric and SI phases	Pepsin, HCl	30 (crop, pH 5.2) + 45 (proventriculus)	40	1.4–2.0	Pancreatin	120	40	6.4–6.8	Broiler chicken	Latorre et al. (2015)
Crop, gastric, SI and caeca	Pepsin, HCl	30 (crop, pH 6.0) + 30 (proventriculus)	40	2.5 (proventriculus), 3.0 (gizzard)	Pancreatin, Sodium bicarbonate, bile salt	120	40	6.2	Broiler chicken	Oliveira et al. (2019)
		60 (gizzard)				20 (caeca)		7.0 (Caeca)		

SI = small intestine; T = temperature.

The artificial gut models that have been used in the past simulated physiological conditions, including pH, temperature, retention time, enzymes and agitation of different compartments of the gut. The mouth and the oesophagus were not included in these models, as no significant digestion occurs in these parts of the GI tract. The artificial gut model generally consists of gastric (proventriculus and gizzard) and small intestinal phases, although a few models also include the crop and the caeca (Oliveira et al., 2019; Suresh et al., 2020). The gastric phase is simulated by exposing the sample, usually ground feed, to hydrochloric acid and pepsin under fixed pH and temperature for an allotted period of time, following by an intestinal phase, where the pH of the resulting slurry is increased through addition of sodium bicarbonate or sodium hydroxide. The sample is subsequently incubated at a fixed temperature and time with pancreatic enzymes, either with or without bile salts as emulsifiers. In the gastric and intestinal phases, feed samples are usually incubated in a shaking water bath at specified revolutions per min, either continuously or for a set time period, although in some cases the sample is only vortexed. Unfortunately, the agitation process has not been described in detail in many studies, hence making it difficult to review and compare this step in the process. Finally, the undigested residues in a filtration unit, or the supernatant after centrifugation, are collected from both the gastric and intestinal phases for analysis. Some gut models, such as those described by Zyla et al. (1995) and Zyla et al. (1999), use an additional dialysis step to simulate nutrient absorption in the small intestine (SI) of poultry. Caecal fermentation steps are carried out separately in most studies, by obtaining aliquots of caecal samples directly from chickens not fed antibiotics and adding them into the final mixture, and then incubating this solution at a fixed temperature, time and pH (Donalson et al., 2008; Dunkley et al., 2007).

Artificial gut models have been used to estimate the digestibility of nutrients, the energetic values of ingredients and feeds, the effect of enzymes, caecal fermentation, changes in gut microflora, and the efficacy of feed additives and adsorbents against mycotoxins (Clunies and Leeson, 1984; Valdes and Leeson, 1992; Bedford and Classen, 1993; Weurding et al., 2001; Donalson et al., 2008; Solís-Cruz et al., 2017; Bryan et al., 2018). Most artificial gut models used in poultry experiments are static. Static models are useful for determining the effect of feed or nutrient composition, feed processing and efficacy of enzymes and feed additives on nutrient bioaccessibility, and representing nutrients that are able to be used by the body. However, static gut models cannot be used to predict interactions between the feed, gastrointestinal environment, and microbiome of the living animal. The gastrointestinal tract of a chicken has a dynamic environment; it adjusts gut conditions in response to the state of digestion, including removal of products of digestion and continuous input of products such as digestive enzymes, acids, bile salts, and ions (Farhadi et al., 2019). The current practices in the use of artificial guts in chickens are presented in the following sections.

### 2.1 Estimation of digestibility

#### 2.1.1 Dry matter and crude protein digestibilities

It is important to determine the dry matter (DM), crude protein (CP), and amino acid digestibilities of ingredients and finished feed regularly, particularly during commercial feed manufacture. Dry matter and CP contents of commonly used feed ingredients can be measured using near-infrared spectroscopy (NIRS), but analysis of nutrient digestibility and determination of undigested nutrients in the GI tract require experiments with animals. An artificial gut can provide a tool for assessing CP and amino acid digestibilities of ingredients that are not published in nutrient databases.

Furuya et al. (1979) developed a two-step in vitro method using pepsin and pig intestinal fluid to simulate gastric and intestinal digestion in swine and other monogastric animals. This in vitro system was used to predict in vivo apparent digestibility of CP and DM in pig diets. The simulated gut developed by Furuya et al. (1979) attracted attention from poultry scientists during the 1980s (Sakamoto et al., 1980; Clunies and Leeson, 1984). Sakamoto et al. (1980) used a similar in vitro method using pepsin and pig intestinal fluid to estimate apparent CP and DM digestibility of poultry diets. When results from the in vitro and in vivo experiments were compared, regression analysis revealed a high correlation coefficient (*r*) of 0.98 and 0.99 for DM and CP digestibility, respectively. The researchers concluded that the in vitro method used was less time consuming, reproducible, applicable to most monogastric animals, and more suited for the evaluation of a large number of samples when compared to the in vivo method.

Clunies and Leeson (1984) investigated the effect of pepsin concentration, time of incubation, pH, and particle size on apparent digestibility of DM and CP, and the relationship between in vitro and in vivo determination of digestibility. They found that digestion of DM and CP was complete after 3 h of incubation at 37 °C, in contrast to 4 h reported by Furuya et al. (1979). This in vitro method provided a good estimation of in vivo digestibility of CP and DM in male broiler chickens at 7 weeks of age (*r* = 0.93 for CP, and 0.99 for DM), when the diets were ground to a particle size of 0.4 mm, which is slightly lower than a particle size of 0.5 mm used by Sakamoto et al. (1980).

There is a scarcity of data available presenting CP and amino acid digestibility rates of commonly used protein sources in chicken diets. Recently, Bryan et al. (2018) proposed an in vitro digestion model mimicking the chicken gut that could be used to predict in vivo digestibility and digestion kinetics of fish meal, porcine meal, soybean meal, corn gluten meal, and corn distillers' dried grains with solubles. The optimum pepsin and pancreatin to substrate ratios for the gastric and the intestinal phases, respectively, was verified using enzyme dose–response assays, with soybean meal as the protein source. The model predicted the rate and extent of protein digestion and presence of undigested protein fractions. However, the researchers highlighted that at least three people were required to collect the samples during the intestinal phase of the assay, and that the resulting digestion constants represent the specific test sample only. In a subsequent experiment by the same group, protein meals (soybean meal, blood meal, feather meal, meat and bone meal, canola meal, fish meal, porcine meal, corn distiller dried grains with solubles) were ranked based on CP digestibility using both in vitro and in vivo analysis (Bryan et al., 2019). The rankings were found to be similar between the in vitro and in vivo analysis, except for corn gluten meal. Following this, Bryan and Classen (2020) performed correlation and regression analysis on the in vitro CP digestibility and in vivo amino acid digestibility data generated by Bryan et al. (2019), and found that in vitro CP digestibility positively correlated with in vivo digestibility of all amino acids except cysteine (*r* = 0.43 to 0.71 for all amino acids, except cysteine). Outputs from these studies indicate that this in vitro assay can be used to classify ingredients based on digestion kinetics values, and to predict in vivo CP digestibility of protein meals used for broiler diets.

#### 2.1.2 Starch digestibility

Understanding rates of starch digestion as an indicator of glucose utilisation by the bird and protein digestive dynamics (Selle and Liu, 2019) is becoming increasingly more important during feed formulation. Thus, it is necessary to regularly monitor starch and CP digestion. An artificial gut may be a useful tool for achieving this, enabling quick measurements of the rate and extent of starch and CP digestion of ingredients used in poultry feed. Furthermore, the data generated from these in vitro assays can potentially be used to develop NIR calibrations for these ingredients.

Starch digestion in chickens occurs in three steps: soaking of feed in saliva and water in the crop, grinding and physical disruption of feed in the gizzard, and chemical hydrolysis and digestion by pancreatic and brush border enzymes in the SI. Weurding et al. (2001) determined starch digestibility in 12 experimental broiler chicken diets with differing starch levels, both in vivo with broiler chickens, and in vitro using a modified version of a simulated SI developed by Englyst et al. (1992) for humans. For the in vitro assay, the diets were passed through a one mm screen and incubated for 30 min, to simulate passage through the proventriculus and gizzard. For the SI phase, incubation periods ranging from 15 to 360 min were investigated, and starch digestion at different time points was correlated with starch digestion in the jejunum and ileum in vivo, to determine the optimum time period. Results showed that for the posterior jejunum a time period of 120 min was the optimum (*r* = 0.94, *P* < 0.05), and 240 min best represented starch digestion in the posterior ileum (*r* = 0.96, *P* < 0.05) section of the SI. There was a significant correlation (*r* = 0.87, *P* < 0.05) between in vitro and in vivo starch digestion rates. The authors concluded that the in vitro method could be used to predict the rate and extent of starch digestion in broilers.

Similarly, Ebsim (2013) established an in vitro assay procedure to determine rate and extent of starch digestion in pea starch compared to other starch sources, using a simulated chicken gut. The gastric phase was similar to that reported by Bedford and Classen (1993), and the SI phase was a modified version of procedures proposed by Englyst et al. (1992), with incubation temperatures and pH selected to closely reflect digestive tract conditions in a chicken. For the SI phase, samples were collected and analysed at 15, 60, and 120 min to reflect digestion in the terminal duodenum, jejunum, and ileum, respectively. The in vitro assay suggested that pea starch is more slowly digested compared to wheat, corn, and barley starch, and that pea cultivar and grind sieve-hole size affect the rate and extent of starch digestion. Although these results were not directly correlated with an accompanying in vivo study, the outputs were in agreement with other in vivo studies, suggesting the potential for practical use of this model. This model was also used by Karunaratne et al. (2018a) to show that wheat market class and cultivar directly impact starch digestibility, and that starch digestibility positively correlates with CP, ash, and non-starch polysaccharides digestibility. In a subsequent experiment, Karunaratne et al. (2018b) observed significant and moderately strong correlations between in vitro and in vivo starch digestibility, but found starch digestibility could not be used to predict apparent metabolizable energy (AME).

In summary, there are multiple examples of artificial gut models being used successfully as alternatives to in vivo tests for estimating nutrient digestibility of poultry diets.

### 2.2 Determination of energetic values of ingredients and feeds

The AME values of feed ingredients and diets are required for establishing, and formulating to, the dietary energy available for utilization by poultry (Hill and Anderson, 1958). However, obtaining these values is time-consuming, labour-intensive and complex. Thus, there is interest in developing in vitro methods for determination of AME values. The AME values of commonly used ingredients in poultry feed can be estimated by NIRS, but NIRS cannot be used to predict the AME values of some alternative ingredients, new ingredients and finished feeds. This is because large volumes of samples are required to build robust NIR calibrations, and AME values of finished feeds cannot be determined accurately using prediction equations for ingredients. Thus, there is an opportunity to develop an in vitro system to measure apparent digestible energy (DE), and use these values to predict AME values of finished feeds.

Clunies et al. (1984) assayed 11 diets to compare the in vitro apparent DE to nitrogen corrected apparent metabolizable energy (AMEn) measured in adult roosters over a 5-d total collection procedure. The results showed that AMEn values of the diets determined in vivo correlated strongly with the apparent DE measured in vitro (*P* < 0.05, *r* = 0.93), with only 4% difference between the two data sets. Only one diet out of the 11 diets presented a fat level (10.5% ether extract) that was notably different between the two assays, illustrating that the in vitro assay may not be successful as a tool for predicting energy digestibility in high-fat diets. When this high fat diet was removed from the analysis, the correlation between in vitro apparent DE and in vivo AMEn further improved (*P* < 0.05, *r* = 0.98). The positive correlations observed in this study are in agreement with a number of other studies which have reported that AMEn in poultry can be predicted from the chemical composition of diets. For example, studies by Carpenter and Clegg (1956) and Sibbald et al. (1963) reported *r* values of 0.949 and 0.953, respectively, when predicting AMEn based on the chemical composition of diets determined using in vitro techniques.

Valdes and Leeson (1992) developed a two-step in vitro system that mimicked gastric and intestinal phases to predict AMEn content in poultry diets. In this experiment, in vivo AMEn values were determined for 71 different poultry diets using adult roosters. The gastric phase used a similar method as proposed by Furuya et al. (1979), except that the particle size of feed was ground to 0.4 mm. The SI phase involved using commercially available pancreatin (trypsin, amylase, and lipase), enterokinase, and bile salts, as replacement for pig intestinal fluid. The in vitro apparent digestibility results showed that only 42.2% of the 71 diets that were tested accurately predicted AMEn, with some ingredients being underestimated (e.g. corn) and some overestimated (e.g. soybean meal, corn gluten meal, and barley) for the rest. This suggests that there may be limited application for use of in vitro techniques as a tool for estimating AMEn values of poultry diets.

Losada et al., 2009, Losada et al., 2010 used a two-step multi-enzyme digestion model proposed for pigs by Boisen and Fernández (1997) to predict AMEn values of starchy grains and cereal by-products, oilseeds, and oilseed by-products for poultry. The predicted AMEn values were ascertained from their chemical composition, in vitro analysis, and NIRS. This in vitro pig model was selected based on its previous success at predicting digestibility and energy values of feedstuffs with high precision in several studies with pigs (Boisen and Fernández, 1997; Noblet and Jaguelin-Peyraud, 2007). However, the researchers found that prediction of AMEn from in vitro organic matter digestibility was not accurate. The model based on the NIRS equation was found to produce the best estimates of AMEn values in the samples.

The simulated gut models used by Clunies et al. (1984), Valdes and Leeson (1992) and Losada et al., 2009, Losada et al., 2010 to predict AMEn values of ingredients and feeds were validated with in vivo data from adult roosters, not broiler chickens. Further, a wide range of ingredients or diets with varying compositions were used to predict AMEn. These limitations were recognized by Yegani et al. (2013), who developed a two-step in vitro gut model to predict AMEn contents of a range of wheat and triticale samples, and validated the results in broiler chickens. The in vitro AMEn results accurately predicted in vivo AMEn values of the wheat and triticale samples for broiler chickens (*r*^2^ = 0.81, *P* < 0.01). The researchers suggested that this provides an opportunity to get quick estimates of AMEn in wheat and triticale, which could be used to develop a calibration database for NIRS scanning. However, Zhao et al. (2014) stressed that the manual handling involved in these experimental procedures, such as pH regulation, digestive enzymes injection, and separation of digested and undigested substance, introduced experimental error, impacting accuracy and reliability of the results. To overcome this, they developed an in vitro computer-controlled digestion system designed to simulate gizzard-intestinal digestion in roosters. The objective of their experiment was to evaluate the effectiveness of this system for predicting AME and true metabolizable energy (TME), using in vitro DE levels determined in rooster diets. The system was designed to control enzymatic digestion conditions automatically, based on enzyme levels determined in the jejunum of 15-week-old roosters (Ren et al., 2012), and clear low molecular weight end products completely at the end of digestion. Results from this study showed that AME and TME could be accurately predicted in most samples using the in vitro DE content of feeds (*r*^2^ = 0.97 for both AME and TME, *P* < 0.001). However, AME and TME predictions for cottonseed meal, rapeseed meal, coconut meal, palm kernel meal, sesame meal, cassava and rice gluten meal were not accurate using this system. Recently, Zhang et al. (2019) used a similar computer-controlled digestion system as the one developed by Zhao et al. (2014) to predict the AME of feed ingredients for ducks. They found that digestible energy determined by the in vitro system could be used to accurately predict TME of feed for ducks.

Overall, there are conflicting results regarding the accuracy of artificial gut models to predict AME values of ingredients and feed for poultry, with success seen in some but not others. The main reason for this is that in complete feed, ingredients are included at various rates resulting in different buffering capacity of the diets. It requires different amount and concentrations of the buffers to be used in vitro system depending on the feed type. Further, for complete feed and feed ingredients, pH, temperature, incubation times, and enzyme concentrations or enzyme type vary depending on the feed or ingredients used. It may be necessary to develop specific methods for specific ingredients or a group of ingredients for successfully estimating AMEn using in vitro methods (Valdes and Leeson, 1992). Further studies are warranted to determine if modifications can be made to these models to make them suitable for determining AME in all feed and feed ingredients.

### 2.3 Assessing the effects of single or multiple enzymes

Enzymes are routinely added to poultry diets to improve digestion and reduce excretion of nutrients. New enzymes need to be screened, categorized and tested in a large number of animals before their efficacy can be determined and they can be released into the commercial market for poultry. Artificial gut models potentially provide an excellent tool for determining enzyme dose rates and efficacy before they are assayed in live chickens.

Bedford and Classen (1993) developed a two-step in vitro system simulating the gut environment, to predict broiler intestinal viscosity and growth when feeding a rye-based diet in the presence of exogenous enzymes. They used commercial pepsin and pancreatin enzymes to simulate gastric and intestinal phases. In this experiment, the in vitro assay accurately predicted in vivo intestinal viscosity (for duodenum and jejunum, *r*^2^ = 0.758, *P* < 0.0001; for ileum, *r*^2^ = 0.667, *P* < 0.0001) and final weight of the birds (*r*^2^ = 0.660, *P* < 0.0001). The researchers concluded that the in vitro assay could be used for rapid screening of enzyme efficacy and optimum inclusion rate for reducing intestinal viscosity and increasing bird weight, without reliance on chickens. Walk et al., 2012a, Walk et al., 2012b used the in vitro digestion assays developed by Bedford and Classen (1993) to investigate the effect of diet, limestone, dicalcium phosphate, phytase, and incubation times on Ca and P solubility. Using this assay, they found that limestone, dicalcium phosphate, and phytase increased P solubility in the gastric phase but decreased it in the SI phase. They also highlighted the importance of pH, retention time, incubation time, particle size, and the use of phytase on Ca and P solubility. However, they did not correlate the in vitro findings with the bird performance. Morgan et al. (2014) used a similar in vitro model and found that Ca and P solubilities in the gastric and SI phases correlated well with in situ findings, although solubility and pH were numerically higher when measured in vitro than in vivo. In this study the in vitro model was unable to determine interactions between the dietary phases, protein source and phytase level on Ca solubility, which the in vivo study could. These researchers concluded that although the in vitro model successfully predicted phytase efficacy, the in vivo study should be performed to investigate the detailed response in the animal.

Zyla et al. (1995) partly adopted the method of Bedford and Classen (1993) when developing an in vitro method to predict P availability in commercial-type turkey diets supplemented with phytase. This assay involved simulating the crop, gizzard, and duodenum. A simulated crop was added in this model because feed can remain in the crop for a longer duration of time in turkeys compared to broiler chickens, which can influence the digestion process. These authors found that the amount of P hydrolysed from feed samples as determined by in vitro digestion correlated strongly with 3-week body weight gain (*r* = 0.986, *P* < 0.0001), toe ash (*r* = 0.952, *P* < 0.0001), feed intake (*r* = 0.994, *P* < 0.0001) and feed efficiency (*r* = 0.992, *P* < 0.0001). The in vitro method predicted P availability in maize-soybean meal based feed containing different concentrations of inorganic P and/or phytase, and thus this method was considered as accurate, cheap, rapid, simple, and robust, and could be easily modified for different species, types of feeds, and nutrients. The researchers used the same method to determine the efficacy of enzyme cocktails and a fungal mycelium in a dephosphorylating corn-soybean meal based feeds fed to growing turkeys (Zyla et al., 1996). It was found that in vitro P release from the experimental diets correlated strongly (*r* = 0.906, *P* < 0.05) with P retention, as also observed in the in vivo feeding trial. Tervila-Wilo et al. (1996) slightly modified the method proposed by Zyla et al. (1995), using different retention times and pH values, to determine digestion of wheat with xylanase and cellulase enzymes from *Trichoderma reesei*. They were able to observe that xylanase and cellulase enzymes degrade feed ingredients cell walls and liberate entrapped protein, but an in vivo experiment was not performed to observe if the efficacies seen in vitro were comparable to that in the bird. Later, Zyla et al. (1999) subsequently modified their earlier proposed turkey model to suit broilers, to examine the effects of xylanase and phytase enzymes on phytate dephosphorylation and arabinoxylan hydrolysis in a wheat-based broiler grower diet. The results showed that the enzyme responses observed in the in vitro experiment were close to those seen in multiple in vivo experiments with similar conditions, although a validation with the exact same diets is lacking. A year later, the same model was used by Zyla et al. (2000) to determine the effects of phosphorolytic and cell wall degrading enzymes on inorganic phosphate, pentoses, protein, and reducing sugar concentrations. This was validated with performance parameters in broilers. There was a correlation between the amount of P hydrolysed from feed samples by in vitro digestion and 3-week body weight gain (*r* = 0.786, *P* < 0.0001). The amount of pentoses in dialysates of feed samples also correlated with feed efficiency (*r* = 0.919, *P* < 0.0001). Similar strong correlations between in vitro analysed phytate hydrolysis and growth performance in poultry in response to phytase addition to diets have been reported in other studies (Morgan et al., 2014; Farhadi et al., 2019). Overall, these studies confirmed that in vitro digestion models can be used to predict in vivo efficacy of phytase on digestion of some minerals including Ca, and P.

Ao et al. (2008) evaluated the enzymatic activities of xylanase, amylase, protease, β-glucanase, and α-galactosidase at different pH levels, simulating pH levels in the crop, gizzard, and proximal and distal parts of the SI. They found that xylanase and β-glucanase were active at a wider range of pH levels compared to that seen with amylase, α-galactosidase, and protease. Ao et al. (2010) studied the effects of citric acid, α-galactosidase, and protease inclusion on in vitro nutrient release from soybean meal using a modified in vitro procedure described by Tervila-Wilo et al. (1996) and Zyla et al. (1999). They found that α-galactosidase and protease enzymes were useful in hydrolysing the carbohydrates and proteins in soybean meal, and that the activity of these enzymes could be increased by acidification of diets with citric acid. Lan et al. (2010) performed two in vitro experiments using the simulated gut model described by Tervila-Wilo et al. (1996) and Zyla et al. (1999) to evaluate the capacity of *Mitsuokella jalaludinii* phytase to hydrolyse phytate in feed. For both in vitro simulation methods, P released from the feed increased quadratically with increasing level of phytase, but the method of Zyla et al. (1995) resulted in more P release than the method of Tervila-Wilo et al. (1996). This is likely because the method of Zyla et al. (1995) used an additional dialysis step which simulated nutrient absorption in the SI of poultry. In a report by Lan et al. (2010), regression analysis showed that the amount of P released in vitro by *M. jalaludinii* could be used to predict in vivo total tract apparent digestibility coefficient of P, CP, DM, and AME.

Menezes-Blackburn et al. (2015) studied the effect of seven commercial phytases on phytate dephosphorylation using the gut model developed by Zyla et al. (1999). The results demonstrated that all phytase products were capable of releasing organically bound phosphate from phytate, but their relative performances were remarkably different. It should be noted that factors such as dietary P levels, intestinal phytase concentration and P absorption were not included in the simulated model. Thus, the researchers concluded that the model cannot be used to rank phytases based on their bioefficacy, as the system cannot fully mimic in vivo situations. However, the simulated model could help reduce the number of feeding trials required when searching for new phytases for poultry applications in the initial screening phase.

Suresh et al. (2019) evaluated the efficacy of xylanase and amylase enzymes produced from *Aspergillus niger* by solid state fermentation, using the simulated gut model described by Latorre et al. (2015). Starter, grower, and finisher feeds, with and without enzymes, were tested. After each digestion step within the gut model, an aliquot of supernatant was collected and analysed for reducing sugar concentration. The enzyme supplemented diets had an increase in reducing sugar concentration compared to the control, indicating the efficiency of these enzymes at improving nutrient availability in vitro. Fengying et al. (2011) also assessed the efficacy of protease, amylase, α-galactosidase, cellulase and pectinase produced by *A. niger zju-Y1* using a simulated poultry digestive tract, and concluded that these multi-hydratases were stable in the gut conditions, so could be used to produce enzymes for poultry feed.

Morgan et al. (2019) investigated the efficacy of xylanase at hydrolysing xylan into xylo-oligosaccharides, both in vitro and in vivo. The in vitro method followed the simulated gut model as described by Walk et al. (2012a), and the in vivo experiment was carried out with broiler chickens. A total of 12 diets varying in soluble and insoluble xylan levels, with and without xylanase, were subjected to the simulated gut model, and the same diets were fed to broilers for the in situ analysis. They found that, for all of the dietary treatments, xylo-oligosaccharide production was highly correlated between the in vitro and in vivo methods, in both the gastric (*r* = 0.85, *P* < 0.05) and SI phase (*r* = 0.92, *P* < 0.05). Thus, the simulated gut model successfully predicted the effect of xylanase supplementation on xylo-oligosaccharide production in the gastric and SI phase in broiler chickens, and can therefore be used to develop non-starch polysaccharide enzymes.

In vitro experiments used to test enzyme effects are strongly correlated with in vivo observations, indicating that an artificial gut may be used to evaluate the efficacy of enzymes for use in poultry.

### 2.4 Evaluating the efficacy of adsorbents against mycotoxins

Mycotoxin binders are routinely added to diets to prevent the toxic effects of mycotoxins in poultry. Numerous mycotoxin binders are commercially available in the market, with varying mode of action. Some primarily target a single mycotoxin while others are more broad spectrum. It is important to test the efficacy and mode of action of mycotoxin binders before applying them to poultry diets.

Solís-Cruz et al. (2017) used the simulated gut model adopted by Annett et al. (2002) and Latorre et al. (2015) to assess the efficiency of four materials (chitosan and three cellulosic polymers) at adsorbing six different mycotoxins. Following the final small intestinal digestion step, the supernatant was filtered and analysed for residual unbound mycotoxins. All four adsorbents had higher binding activities against all the mycotoxins compared to the control without adsorbents. The cellulosic polymers showed a better adsorbent capacity for all the mycotoxins compared to chitosan. Among the cellulosic polymers, sodium carboxymethylcellulose and microcrystalline cellulose were identified as the optimum candidates for binding mycotoxins.

Zavala-Franco et al. (2018) used the gut model of Solís-Cruz et al. (2017) to evaluate the efficacy of three biosorbents against aflatoxin B1 contaminated poultry diet. A positive control diet with zeolite (natural aflatoxin binder) was also used, to compare the effectiveness of biosorbents and zeolite against aflatoxin B1. The results showed that the addition of biosorbents and zeolite to the diet significantly reduced the bioavailability of aflatoxin B1. Of the three biosorbents investigated, Aloe powder was found to be the most effective aflatoxin B1 binder.

Tso et al. (2019) studied the effects of seven commercial mycotoxin removers (two adsorbents and five enzyme degradation reagents) against two mycotoxins (deoxynivalenol and zearalenone) using an artificial gut model similar to that of Zyla et al. (1995). The results suggested that the enzyme degradation reagents were more effective in removing deoxynivalenol and zearalenone compared to the adsorbents. Kolawole et al. (2019) designed an artificial gut model to assess and compare the efficacy of 10 commercial mycotoxin binders and found that only a modified yeast cell wall product effectively adsorbed more than 50% of the mycotoxins, whereas the others were only moderately effective.

The artificial gut models used in the above studies successfully screened the elite mycotoxin binders for use in poultry. However, none of the above studies compared the results with in vivo experiments, so it is unclear if the findings generated by the artificial gut would translate to improved performance in chickens.

### 2.5 Determining the efficacy of feed additives and estimation of caecal fermentation

The ban on use of in-feed antibiotics in many parts of the world has resulted in the re-emergence of poultry diseases that were effectively controlled in the past. Consequently, efforts to find alternatives to in-feed antibiotics are ongoing, with a big focus on alternate nutritional strategies that improve gut health. Some of these alternative feed additives include probiotics, prebiotics, synbiotics, organic acids, essential oils, plasma proteins, phytochemicals, and functional fibres. There are a large number of commercial feed additives in the market with different claims and modes of action. Experiments are needed to determine the efficacy, safety, usage rate, and environmental impacts of these additives before they can be used in poultry. Artificial gut models may provide a tool for testing the efficacy of these additives and categorizing them before they are selected for in vivo experiments.

Chang and Chen (2000) studied the impacts of a *Lactobacilli* mixture on *Campylobacter jejuni* in an artificial gut model, and found that the *Lactobacilli* mixture reduced the *C. jejuni* population in the gizzard and SI section. Donalson et al. (2008) investigated the effects of dietary supplementation of a fructooligosaccharide prebiotic with alfalfa on caecal fermentation in laying hens. The results showed that fermentation of alfalfa produced more acetate, lactic acid, and volatile fatty acids compared with the layer diet alone. The addition of a fructooligosaccharide prebiotic resulted in increased fermentation, with maximum values obtained after 24 h. Dunkley et al. (2007) used a similar in vitro cecal fermentation method, and showed that high fibre sources increased fermentation and microbial diversity in the caeca.

Meimandipour et al., 2009, Meimandipour et al., 2010 evaluated the potential of two strains of *Lactobacillus* on short chain fatty acid production and quantification of butyrate-producing bacteria in a simulated broiler caecum. The results showed that *Lactobacilli* supplementation inhibited the growth of *Salmonella* and increased the population of *Lactobacilli*, *Bifidobacteria*, and butyrate-producing bacteria *Faecalibacterium prausnitzii*, but had no effect on *Escherichia coli* numbers. Further, lactate was only detected in the treatments containing the two *Lactobacillus* strains. The study highlighted that the two strains of *Lactobacilli* could potentially be used as a probiotic dietary supplement for broilers.

Wali and Beal (2011) investigated the survival of *Lactobacillus salivarius NCIMB 41606* in an artificial gut model, and its effect on the survival of *Salmonella typhimurium NAL*^*R*^ *SALL344*. The study found that *L. salivarius* survived through the artificial digestion process, and the presence of *L. salivarius* did not affect the survival of *S. typhimurium* compared to the control. Furthermore, Robyn et al. (2012) simulated the broiler chicken caeca to screen lactic acid bacterial strains capable of inhibiting *C. jejuni*, and Card et al. (2017) used an artificial caecal fermentation model to examine the effect of *Salmonella enterica* serovar Typhimurium on caecal microbiota and the impact of antibiotic administration. The model helped to estimate chicken caecal microbial diversity and served as a powerful screening tool to evaluate and refine interventions to mitigate the spread of antibiotic resistance in the gut.

Latorre et al. (2015) employed the in vitro digestion model designed by Zyla et al. (1995) to investigate the effect of a selected *Bacillus* spp. on digesta viscosity and *Clostridium perfringens* (Cp) counts in a broiler diet containing corn, wheat, rye, barley, and oat. In this study the supernatants were collected from each simulated gut section. The results revealed that the dietary inclusion of the *Bacillus* spp. reduced viscosity and Cp counts compared with the control diets. This in vitro study produced preliminary data on the efficacy of *Bacillus* spp., and highlights that further research is warranted into its effects in broilers challenged with pathogens such as necrotic enteritis.

Oliveira et al. (2019) used an artificial gut model to investigate antimicrobial effects of essential oils and organic acids against *Salmonella enteritidis*. Crop, gastric (proventriculus and gizzard), SI, and caecal phases were simulated, covering a total duration of 4 h and 20 min. The results showed that the additive mixture inhibited the growth of *S*. *enteritidis* across all phases, but did not affect the population of *Lactobacillus plantarum* significantly. Suresh et al. (2020) investigated pyroligneous acid as a therapeutic agent against *Salmonella* using an artificial gut model which included gastric, SI, and caecal fermentation phases. The results indicated that the dietary supplementation of pyroligneous acid at 1.6% inhibited the growth of *Salmonella*, and had no negative effect on *Lactobacillus* counts or the production of acetic acid in vitro.

Efforts should be made to develop a dynamic gut model for poultry. A dynamic gut model has provisions for controlling gastric and intestinal transit times and flow rates, mixing of gastric and SI contents, maintaining the composition of digestive fluid and pH values, and removal of water and digested compounds. The dynamic gut models used for humans and other animals include the dynamic gastric model (DGM), human gastric simulator (HGS), the artificial colon (ARCOL), DIDGI system (gastric and SI model), TIM-1 (gastric and SI model) and TIM-2 (large intestine model) simulator, the human intestinal microbial ecosystem (SHIME), the engineered stomach and small Intestinal-ESIN-system, and the SIMulator of the Gastrointestinal tract (simgi) (Dupont et al., 2019). However, the GI characteristics of poultry are different to that of humans and pigs, mainly due to the presence of a gizzard and pair of caeca, as well as differing intestinal temperatures and pH values, meaning these models do not necessarily apply to poultry research. Efforts are underway to develop a dynamic artificial gut model for chickens. Evonik, a German company, and ProDigest, a Belgian company have been working in this area (Evonik, 2017a, Evonik, 2017b). However, no literature has demonstrated the application of such systems in poultry to the best of our knowledge. Thus, there is an opportunity to develop a dynamic artificial gut model for poultry research and production purposes.

## 3 Conclusion

An artificial gut should simulate gastric digestion, SI digestion, and caecal fermentation, with physiological conditions (temperature, pH, retention times, agitation frequency) mimicking the in vivo gut environment as close as possible. A wide range of artificial gut models have been developed and used in poultry research. However, most of these models are static. These static gut models should be standardized and validated to achieve reproducible results, and to ensure results are consistent among laboratories. Ideally, an artificial gut model should be dynamic, with provisions to clear low molecular weight end products completely at the end of digestion. From this review, it is plausible to think that an artificial gut model can be developed for poultry that can simulate the whole GI tract and works as well as the human models. The model should be automated and with less manual procedures to minimize errors. Scientific efforts should be directed towards development of a standard artificial gut model for poultry. This would be a leading step towards the implementation of the 3Rs animal ethics principles (Replacement, Reduction and Refinement) in poultry research, and provide potential to perform large scale assays of poultry feed and additives prior to feeding to live animals.

## Author contributions

**Nishchal K. Sharma**: conceptualization, original draft preparation; **Shu-Biao Wu**: reviewing and editing; **Natalie K. Morgan**: reviewing and editing; **Tamsyn M. Crowley**: conceptualization, reviewing and editing.

## Declaration of competing interest

We declare that we have no financial and personal relationships with other people or organizations that can inappropriately influence our work, and there is no professional or other personal interest of any nature or kind in any product, service and/or company that could be construed as influencing the content of this paper.
